# Current state of populations of *Rhodiola rosea* L. (Crassulaceae) in East Kazakhstan

**DOI:** 10.1186/s40529-021-00327-4

**Published:** 2021-11-07

**Authors:** Serik A. Kubentayev, Moldir Zh. Zhumagul, Meruyert S. Kurmanbayeva, Daniar T. Alibekov, Jurii A. Kotukhov, Gulnara T. Sitpayeva, Saule K. Mukhtubayeva, Klara S. Izbastina

**Affiliations:** 1 «Astana Botanical Garden» branch of the Republican State Enterprise on the right of economic management “Institute of Botany and Phytoinroduction”, Nur-Sultan, Kazakhstan; 2 Al-Farabi Kazakh National University, Almaty, Kazakhstan; 3 Republican State Enterprise “Altai Botanical Garden”, Ridder, Kazakhstan; 4 Republican state enterprise on the right of economic management “Institute of Botany and Phytointroduction” of the Committee of Forestry and Wildlife of the Ministry of Ecology, Geology and Natural Resources of the Republic of Kazakhstan, Nur-Sultan , Republic of Kazakhstan

**Keywords:** Ontogenesis, Kazakh Altai, Asia, Chorological analysis, Life forms, Morphological and quantitative indicators, Conservation, *Sedum roseum*

## Abstract

**Background:**

Based on world experience, first, a modern assessment of the flora is needed to develop strategies for the conservation of ecosystems of rare and endangered plant species. A regional and global biodiversity strategy should focus on assessing the current state of bioresources. To preserve the biodiversity of the species and its habitat, we evaluated botanical features, ontogenetic phases, the ecological and phytocenotic structure of the rare and endangered of *Rhodiola rosea* L. (golden rose root) populations from the highlands of Eastern Kazakhstan.

**Results:**

*R. rosea* in the study region lives on damp mossy rocks, rocky slopes, overgrown moraines and along the banks of mountain rivers in the upper limit of cedar-larch forests, subalpine and alpine belts, in the altitude limit of 1700–2400 m. In the studied region, *R. rosea* begins to vegetate in May–June, blooms in June–July, the fruits ripen in August. The species is encountered in the high mountain ranges of the Kazakh Altai and Saur-Tarabagatai. Unfavorable habitat conditions for the species are overgrown by sedge-grass and birch-moss communities. The most common species at sites with *R. rosea* are: *Schulzia crinita, Achillea ledebourii, Doronicum altaicum, Macropodium nivale, Hylotelephium telephium, Rhodiola algida, Carex capillaris, C. aterrima.* Ontogenetic study revealed that all age-related phases were present, with the exception of the senile states. Individual life expectancy shown to be 50–55 years. The analysis of the species composition in the communities with *R. rosea* showed that the leading families in terms of the number of accompanying species are *Poaceae, Ranunculaceae, Asteraceae, Rosaceae and Caryophyllaceae, Apiaceae, Fabaceae*; while the most dominant genera are: *Carex, Aconitum, Dracocephalum, Festuca, Pedicularis, Poa, Salix*; the ecological groups are dominated by psychrophytes, mesophytes mesopsychrophytes; the Asian, Eurasian, and Holarctic groups are the most represented groups. Dominant life forms according to Serebyakov were rod-rooted, brush-rooted, short-rooted and long-rooted grasses, while based on Raunkiaer’s groups the overwhelming majority consisted of Hemincryptophytes (74%).

**Conclusions:**

The *R. rosea* populations of Kazakhstan represent an important gene stock of the species. Our study provides new insights into the species’ biology thus contributes to the conservation of biodiversity on a wide spatial scale.

## Background

The study of the ecological and botanical characteristics of natural populations of rare and vulnerable plants remains a priority in the strategy of biodiversity conservation, especially if they are Wild Relatives of crops (Perrino and Wagensommer 2021). Currently, many valuable medicinal plants are subjected to spontaneous gathering, as a result of which the number and areas of natural habitats are decreasing, the natural balance in communities is disrupted, which leads to population degradation (Cunningham et al. 2020).

This concerns *R. rosea*, the demand for which has grown significantly throughout the world in recent years, which threatens the extinction of natural populations on a global scale. The global demand for *R. rosea* raw material will continue to grow (Bernard 2016), which could lead to catastrophic consequences. The main drivers of the increased demand for *R. rosea* raw materials are the expansion of the range of drugs, dietary supplements, and cosmetics containing *R. rosea* (Brinckmann et al. 2020). A highly important priority is to preserve the natural environment in which *R. rosea* grows and it is a high priority is to evaluating the type and flock size of the grazing in order to preserve their natural habitat, using sustainable criteria (Perrino et al. 2021; Buse et al. 2015). Nowadays *R. rosea* is classified as rare and endangered species, in many regions—as a protected plant. The growing demand increases the load on the natural populations of the species. As a result, it has become a threat object in many Eurasian countries, including the Czech Republic (Grulich 2012), Slovakia (Ferakova et al. 2001), Bosnia and Herzegovina (Platikanov and Evstatieva 2008), Bulgaria (Tasheva and Kosturkova 2012), Germany (Metzing et al. 2018), Austria (Niklfeld and Schratt-Ehrendorfer 1999), the Russian Federation (Trutnev et al. 2008), Mongolia (Urgamal 2018), China (Qin 2017).

One reliable way to preserve this species is to introduce it into cultivated culture (Karpukhin and Abramchuk 2020). The scientists suggest that in situ protection is insufficient to preserve the gene pool of the *R. rosea* population. It seems appropriate to create ex situ populations and return them to nature (Hou and Lou 2011). It is successfully cultivated in botanical gardens and in research institutes of plant growing in Russia (St. Petersburg, Gorno-Altaisk, Novosibirsk, Irkutsk, etc.) (Moryakina et al. 2008) as well as in European countries: Bulgaria (Platikanov and Evstatieva 2008), the United Kingdom (Peschel et al. 2018), Poland (Buchwald et al. 2015), Italy (Fusani 2019).The regenerative capacity of wild plants is limited due to the very low seed germination rate (5–35%) and the coefficient of vegetative reproduction (Platikanov and Evstatieva 2008). In view of the above, the widespread use of natural specimens in many countries has led to the disappearance of the species, which has provoked the adoption of a number of conservation measures: cultivation in appropriate conditions (Matthys et al. 2007). In many countries, sustainable ecological use of natural resources, the conservation and the preservation of natural areas as a special environmental activity are regulated (Yaneva et al. 2020).

Despite the great interest in the *R. rosea* and extensive research in the field of phytochemistry, plant bio-technology remained less researched and widely used (Li et al. 2019; Olsson et al. 2009). The stages of morphogenesis of the species and the maximum age of wild populations of *R. rosea* are also poorly studied (Brinckmann et al. 2021). The studies of many Russian scientists are devoted to the study of ecological and botanical proper-ties, distribution, ontogeny of populations (Sofronov et al. 2016; Valuiskih et al. 2017; Panossian et al. 2010; Yakubov et al. 2019; Shadrin et al. 2020). A relatively fewer population ecological studies of *R. rosea* were performed in Europe and North America (Olfelt et al. 2014; Aiello et al. 2013), however, the research on genetic diversity and phylogeography of populations are well represented (György et al. 2012; György et al. 2013; György et al. 2014; Soni et al. 2010; Kozyrenko et al. 2018).

*Rhodiola rosea* L. (Crassulaceae DC) has a wide circumpolar distribution in the northern hemisphere from the low-Arctic to high-temperature regions of Asia, Europe, and North America (Cuerrier 2014; Brinckmann et al. 2021). It is a rare species included in the Red Book of Kazakhstan (2014) with III class status and is regarded as a threatened species. According to the data of International Union for the Conservation of Nature Resources (IUCN), the rarity category is Least concern (LC) (Chadburn 2020). It is guarded in Katon-Karagai State National Natural Park, Markakol and Eastern Altai protected area in the researched region. It grows in alpine and subalpine vegetation belts, stony tundra’s, on the rocks and rocky hills, on placers and moist soils along river banks. In Kazakhstan, it has been documented in three floristic regions: Altai, Tarbagatai, Dzhungarskiy Alatau (Vasilyeva 1961). Distribution area is in the Southern Siberian mountains, in the Ural, in transpolar regions of Yakutia, in the highland areas of Eastern Siberia and Far East, on the coasts of White and Barancevo seas, in Mongolia, China, North America and in Asia Minor (Borisova 1939; Vasilyeva 1961; Ivanova 1979; Peshkova 1994).

Therefore, purpose of the work is to study distribution and density of *R. rosea* populations in East Kazakhstan, floristic and habitat characteristics, including variation in ontogenetic states of the individuals.

## Materials and methods

The Kazakhstan part of Altai is a system of ridges in the southern and the southwestern part of Altai, as a mountainous country that stretches from south to north and from west to east for almost 400 km. It is a part of the southwestern periphery of the Altai-Sayan mountain system and with its inherent structure of landscape and high-altitude zones. According to the physical and geographical conditions, the territory of the Kazakhstan Altai is subdivided into three subdistricts which are Southwestern Altai, Southern Altai, Kalbinsk Highlands (Yegorina et al. 2003).

The research was carried out between 2015 and 2020 in Southern Altai (Narym, Sarymsakty, Southern Altai Tarbagatai, Kurchym ridges) and Western Altai (Ivanov, Ubi, Ulbi, Koksim Linei, Western Listvyaga ridges) of Kazakhstan part. The investigated region administratively belongs to the East Kazakhstan region. The geographic zoning and names of mountain ridges are specified according to the Physical Map of Kazakhstan. To identify the phytocenotic features of *R. rosea* populations, the traditional methods of field geobotanical studies were perfumed using the ecological-physiognomic approach. The ecological and physiognomic types combine plant communities with dominants belonging to one ecobiomorph and ecologically similar groups of species (Bykov 1970).

The description of the populations was carried out using special description forms. In each population, 15 study sites were laid, the area of the site was: 10 × 10 m (100 m^2^). A total of 150 sites were taken into account. The GPS device recorded the marginal points of the community boundaries to identify the area. First, general information was written in the form: description number, geographical location, date, coordinates, height, site size, photo number, then the following main sections are reflected in the form: The name of the vegetation type based on dominant species; the floral composition of the community with an indication of occurrence of species. To do this, we selected a section in the redevelopment of a homogeneous contour. The GPS device determined the coordinates and the absolute height.

To calculate the frequency of species in the floristic composition of *R. rosea* communities, all plant species were accounted for in each surveyed accounting site, the obtained values were grouped into five occurrence classes: I-0-20%, II-21-40%, III-41-60%, IV-61-80%, V-81-100%.

The rarity category and status of the species are indicated in accordance with the Kazakhstan Red Data Book (Kazakhstan Red Data Book 2014) and The IUCN Red List of Species (IUCN 2020). Uranov (1969) method was applied for the purposes of researching the life cycles. The methodological guidelines developed by Golubev and Molchanov (1978) were used as a basis for studying ecological, biological properties of the species in the real-life field conditions.

The spectra analysis of geographic elements of floras of various ranks, including floras of plant communities in the scope of specified classification units (composition of the floras) is one of the main tools of comparative floristical studies. Composition of the flora is a set of plant species which form communities of any rank and any type of vegetation. From this point of view, the composition of the flora represents the unification of historically and coenotically homogeneous groups of species within the syntaxon, which makes them the most important indicator of the vegetation cover from the level of a particular phytocenosis to altitudinal-belt units. The life forms of plants of the floristic composition of *R. rosea* communities are given according to the classifications of Serebryakov (1962) and Raunkiaer (1934). Species composition in *R. rosea* communities by ecological groups and area of species are given according to the classification of Kuminova (1960).

Plant names are listed according according to Plants of the World Online (POWO 2021). The analysis of species’ floral composition in the surroundings of *R. rosea* was carried out in comparison with the Alpine flora of Altai (AFA) (Revushkin 1988).

The studies were conducted according to the scheme proposed by Rabotnov (1964) and Smirnova (1976). The following classification of the age groups were used in the description: plantlets (p), juvenile (j), immature (im), virginile (v), young generative (g1), mature generative (g2), old generative (g3), ageing individuals (a.i.). " Distribution map of *R. rosea* in East Kazakhstan” was obtained by ArcMap.

According to the number of fibers remaining annually after the death of the shoots on the *R. rosea* rhizome, it is possible to judge the age of plants. In any cenosis, as a rule, all species are represented by numerous individuals of various ages, from seedlings to old plants. We studied all individuals of the species in different populations and established the course of accumulation of fibers with age, and based on this we calculated the age of individuals. Undoubtedly, the determination of the age of individuals by this method may be inaccurate, since some of them are characterized by a very slow course of development, while others, on the contrary, develop very quickly. However, the accuracy of age determination increases with an increase in the number of studied individuals. We determined the age of 100 individuals from different populations.

**Fig. 1:**
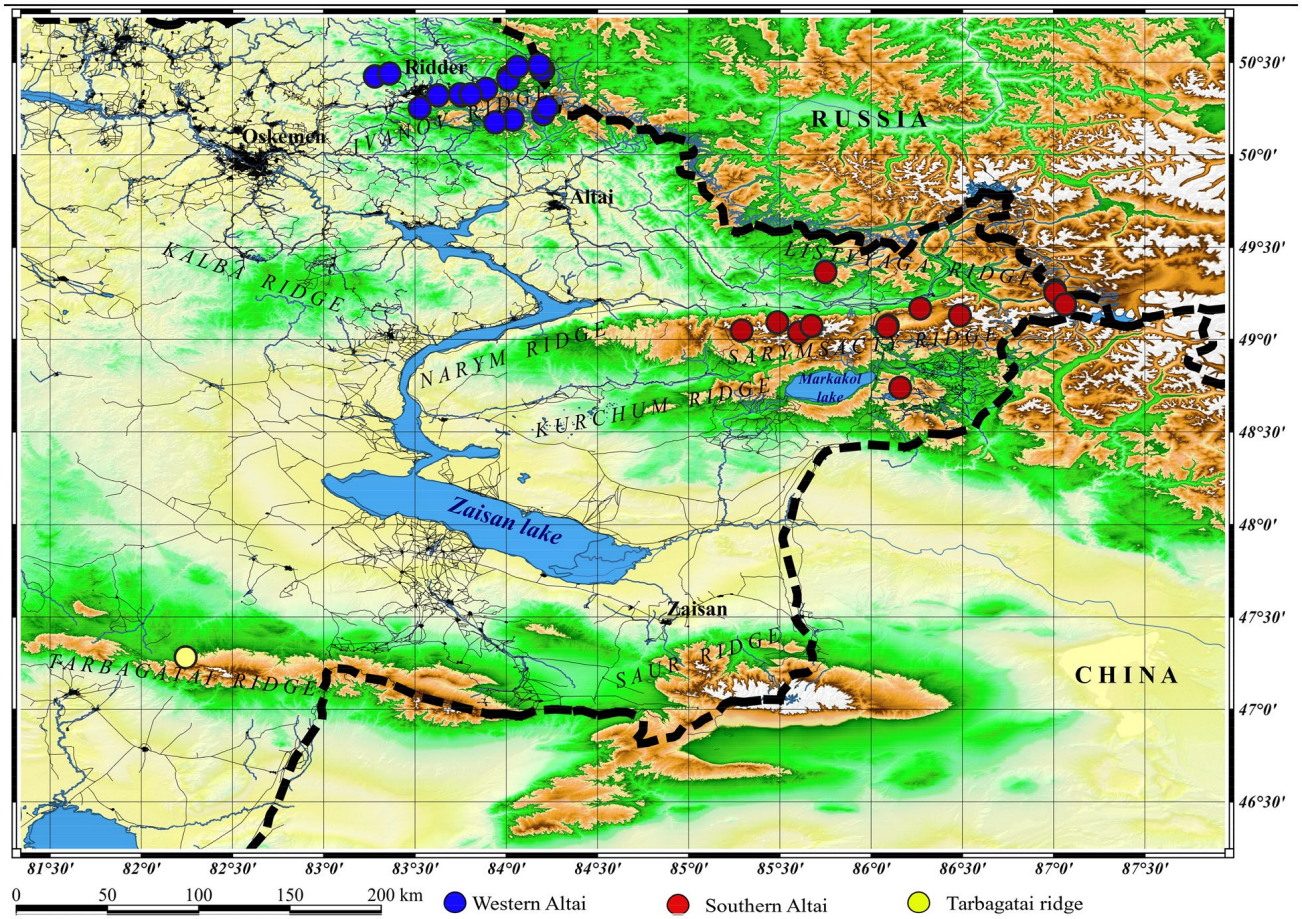
Scheme map of the distribution of the *R. rosea* in East Kazakhstan (Scheme map was obtained by ArcMap)

Based on the long-term herbarium collections of the authors of this work stored in Altai Botanical Garden (further referred to as ‘Alt.’) and Astana Botanical Garden (NUR), as well as a result of the revision of the herbarium materials of the Moscow State University (MW) (Seregin 2020) and the herbarium of the Institute of Botany and Phytointroduction (AA), the distribution of *R. rosea* in Eastern Kazakhstan was revealed. In addition, the following literature were taken into account: Perrino et al. 2021; Artemov 2020; Kotuhov 2005; Isayev 1993; Zibzeyev and Nedovesova 2015; Kubentayev et al. 2018, Kubentayev et al. 2019. The distribution map is shown in Fig. 1.

The correlation analysis was calculated using the Rstudio software (Rstudio Team 2015). Statistics of morphometric parameters were performed using the program Statistica 10 (StatSoft STATISTICA 10 2011).

## Results

### Distribution of *R. rosea* in East Kazakhstan

*R. rosea* is distributed on the ridges of the Kazakhstan Altai and Tarbagatai in the East Kazakhstan region. The species is found in the following administrative districts: Katon-Karagaysky, Kurchumsky, Riddersky, Glubokovsky and Urdzharsky districts. The examined herbarium samples are given below.

Specimens examined: WESTERN ALTAI: was revealed: Ivanovski ridge: vicinity of Ridder mountain, Khorizovka river narrow, 29.VI.1936, Rubam & Mikhailova (АА); near the small area, between blocks of moraine debris, 15.VI.2015, Kubentayev (NUR); top of Poperechka river, along the river bank, 03.VII.2012. Kotukhov (Alt.); «Prohodnoj belok», along the damp rocky places, 10.VI.2016, Kubentayev (NUR); vicinity of Rodonovoi river, along the damp rocky places, 10.VI.2015, Kotukhov (Alt.); Ulbi ridge (northern faces of Kreslova mountain, Ridder region, 26.VII.1937, Kuznecov (АА, MW); Lineiski ridge: valley of Chernaya Uba river, south- western slope on the altitude of 1830 m., 03.VII.1998, Kotukhov (Alt.); Koksi ridge: in Latuniha river-valley, 27.VI.2003, Kotukhov (Alt.); in Chernaya Uba river-valley, 03.VI.2003, Kotukhov (Alt.); Ulbi ridge: Bolshoi Turgusun river-valley, 15.VI.2004, Kotukhov (Alt.); top of Tatarka river, 15.VII.2004, Kotukhov (Alt.); Ubi ridge: Belaya Uba river-valley, 12.VI.2006, Kotukhov (Alt.). IN SOUTHERN ALTAI: Chindagatui mountains: Southern Alai ridge, moist meadow in the lower part of the slope, 1800 m., 27.VII.1986, Ivaschenko & Utebekov (AA); Narym ridge: Koksar mountain, 7.VII.1973, Mikhailov & Stepanova (AA); Southern Altai-Tarbagatai ridge: Burkhat pass, northern slope at the top of the forest, 14.VII.1973, Isayev (АА); left bank of Karakaba river, northern slope, 2100 m., 28. VI.1988, Ivaschenko (АА); vicinity of Chernovoye village, in the upper part of the forest, 15.VI.2016, Kubentayev (Alt.); in the vicinity of the bridge across Karakaba river along the Austria road, damp rocky places, 18.VI.2016, Kubentayev (NUR); to the southern-east of Enbek village, at the top of a ridge in the alpine belt, 01.VI.2017, Kotukhov (Alt.); Sarymsakty ridge: Kumshybai, by the stream along the path to the waterfall, damp meadows, 22. VI.1986, Ivaschenko (AA); at the top of Solonechnaya river, 22.VIII.2010. Kubentayev & Zhumagul (NUR); in the vicinity of Topkain river, alpine meadows, 10.VIII.2020. Kubentayev (NUR); Western Listvyaga ridge: at the foot of Schebniuha hill, valley of mountain river, 10.VII.2019, Kubentayev (NUR); Repnoie river head, Kubentayev & Zhumagul, 22.VII.2019; vicinity of Aksharbak village, Katon-Karagai region, Verhkatun riverhead, 22.VII.2020. Kubentayev (Alt.).

### Ecological–biological and phytocenotic structure of *R. rosea* populations

**Fig. 2:**
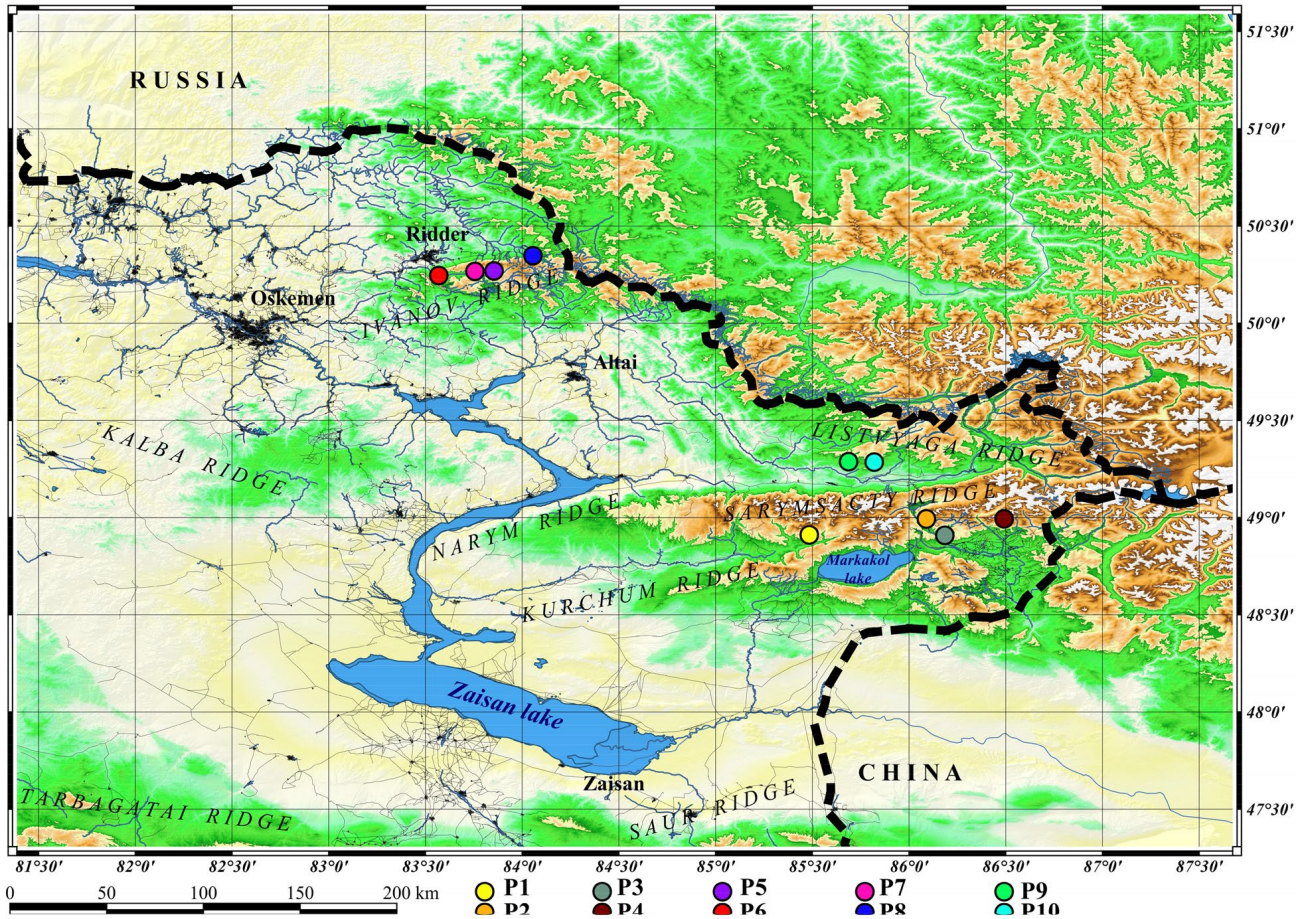
Geographic locations of the studied *R. rosea* populations

The study of *R. rosea* populations was carried out at 4 geographic sites: Ivanov ridge (4 population); (2) Sarymsakty ridge (2 Population); (3) Southern-Altai Tarbagatai ridge (2 Population); (4) Western Listvyaga ridge (2 Population) belonging to the territory of Kazakhstan Altai (22. Altai), according to the floristic zoning of Kazakhstan (Kazakhstan Flora 1957). The species populates wet moss-covered rocks, rocky hills, near snowfields, overgrowing morains, among moss along the river banks, in the upper limit of mossy cedar-larch forests (Fig. 2).

**Fig. 3:**
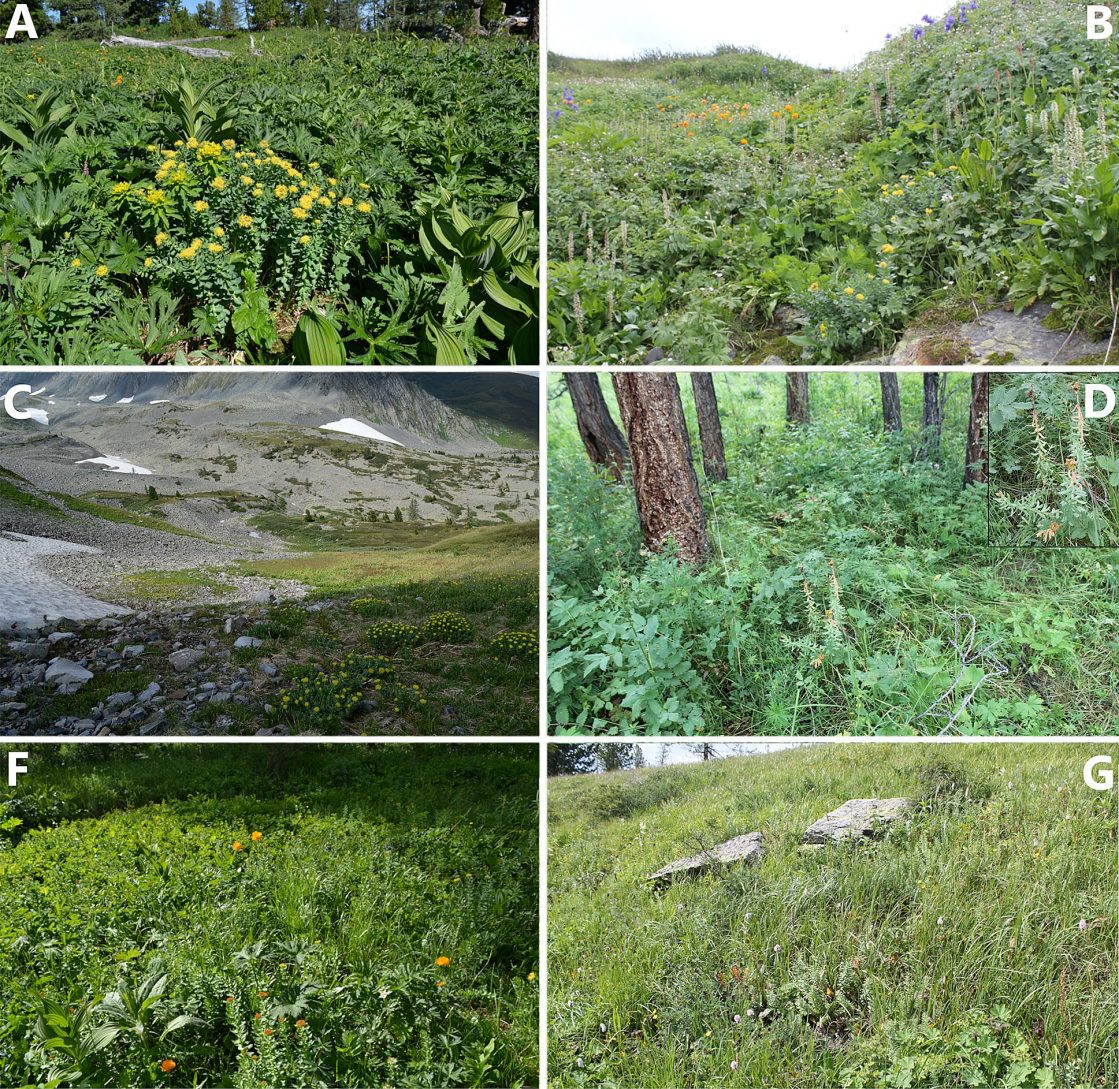
Populations of *R. rosea* in different ecological conditions (**A** in damp alpine meadows; **B** in shallow shallow riverbeds; **C** on weakly overgrown moraines near glaciers; **D** in cut cedar-larch forests; **F** in motley grass alpine meadows; **G** in heavily overgrown herb-grass areas (degrading populations)

1. Population (*Macropodium nivale –A ngelica archangelica *– *R. rosea* ) is timed to the western slopes of shallow ravines of shallowed river beds (Fig. 3). The population was studied at Burkhat pass, Sarymsakty ridge, (49°07’49.9"N, 86°02’19.8"E) in the altitude of 1950–2050 m. Projective cover (PC) is 55%. Standing grass is formed diffusively, along the rock cracks, between the debris of blocks and in degradations where the fertile soil layer accumulates. Recorded accompanying species: *Coptidium lapponicum, Aquilegia glandulosa, Sanguisorba alpina, Rumex acetosa, Bistorta elliptica, Trollius altaicus, Geranium albiflorum* and etc. in the community. The population of *R. rosea* as a rule, is represented by all age classes with predominance of generative adult specimens. The state of this population of *R. rosea* is characterized as stable, capable of self-renewal.
2. Population (*Carex stenocarpa + C. orbicularis + R. rosea + Dracocephalum grandiflorum*) occurs in well overgrown steep moraine slopes, Sarymsakty ridge, Solonechny river (49°05’34.7"N, 85°29’10.3"E). Moraine hillocks are located on the steep northwestern slopes of the ridges in the altitude of 1900–2100 m above the sea level with closely located snowfields. PC is 80–90%. Typical species for these communities are *Festuса kryloviana, Allium schoenoprasum, Hedysarum neglectum, Anemonastrum narcissiflorum, Thalictrum alpinum, Papaver nudicaule, Neogaya simplex, Gentiana grandiflora, Pedicularis oederi, P. amoena, P. violascens, Oxytropis alpine*, rare enough *Gentiana algida, Papaver nudicaule, Festuca altaica. Betula rotundifolia* should be noted among the shrubs, which forms separate small patches. Favorable water and temperature conditions, high humus content in the soil determine the lush development of vegetation, which negatively affects the populations of *R. rosea* due to its low competitiveness.
3. Population (*Alchemilla gottsteiniana – Polygonum ellipticum *+ *R. rosea* ) is timed to smooth slopes of alpine meadows on the meadow soils (Fig. 3), Southern Altai Tarbagatai, Karakaby depression, Kara-Kaba river-valley (49°04’06.8"N, 86°05’14.8"E). Total PC is 85%. The shrub layer is poorly expressed, *Lonicera altaica, Potentilla glabra* is rare *Spiraea media* shrubs are only noted. *Koenigia alpina, Phleum alpinum, Valeriana dubia, Rumex acetosa Galium boreale, Iris ruthenica, Dracocephalum grandiflorum, Aster alpinus, Papaver nudicaule, Vicia cracca, Sedum hybridum, Pedicularis achilleifolia, Pachypleurum alpinum, Oxytropis alpina, Gentiana algida, Crepis chrysantha* are frequently often encountered in the phytocoenosis. *R. rosea* population in this type undergo degradation. Gradually expanding, meadow vegetation displaces *R. rosea* from familiar habitats. Ontogenesis is dominated by generative and senile individuals, reproduction by seeds is absent.
4. Population (*R. rosea* + *Anemonastrum narcissiflorum – Сагех aterrima – Thermopsis alpina – Viola biflora*) occupy excessively cold, moderately humid rocky peaks and southwestern slopes of weakly closed pressure moraine ridges in the altitude limit of 2000-2300 m. above sea level, Southern Altai Tarbagatai ridge (49°10’04.4"N, 86°16’07.3” E). PC is 30 %. The vegetation cover is less developed and relatively poor in terms of species. The most common herbaceous plants are, *Carex capillaris С. orbicularis, C. rupestris, Schulzia crinita, Micranthes punctata, Papaver croceum, Salix rectijulis, Dryas oxydontha, Silene graminifolia, Koenigia alpina, Minuartia verna, Patrinia sibirica* is relatively common among shrubs. This *R. rosea* population is represented mainly by aging generative and very old individuals. Analysis behind the developmental state of *R. rosea* in the upper limit of its distribution suggest considering grounds to consider these habitats as extreme.
5. Population (*Hedysarum neglectum + Carex orbicularis + C. aterrima *– *R. rosea* ) occurs in weakly covered moraine ridges (Fig. 3), Ivanov ridge, upper parts of Big Poperechka river (50°19’13.5"N, 83°45’11.0"E). Plants are usually along the northwestern microslopes, on the altitude of 2000–2300. PC is 70%. Species observed here were *Rhodiola algida, Dryas oxyodonta, Bergenia crassifolia, Hedysarum neglectum, Pedicularis achilleifolia, Neogaya simplex, Oxytropis alpina, Pedicularis amoena, P. oederi*, in the community *Hedysarum theinum, Saussurea alpina, Schulzia crinita, Luzula spicata* occurs comparatively rare. Open areas are densely covered by *Polytrichum juniperinum, Р. аlpinum* mosses and species from the genus *Bryo*. Patches of lichens from the genus *Cladonia* are commonly encountered. *R. rosea* in the coating occupies no more than 5–6% of the total surface.
6. Population (*Deschampsia caespitosa + Senecio pratensis* + *R. rosea*) observed on moderately humid stony-mobile fine-gravel slopes of moraine ridges, Ivanov ridge, passing snow-covered mountain peak (50°15’10.3"N, 83°31’31.8"E). It is encountered on the altitude of 1800-1900 m above sea level. PC is not more than 40%. In this kind of conditions *R. rosea* grows far from drains, constantly face the lack of moisture. The vegetation cover is dense, accompanying species are: *Carex pediformis var. macroura, С. capillaries, Festuca borissii, Helictotrichon altaicum, Lagotis globosa, Callianthemum alatavicum, Saussurea alpina, Dracocephalum grandiflorum* here in the community. *Dryas oxyodonta, Thalictrum alpinum, Gentiana algida, Eritrichium villosum, Allium schoenoprasum, Pachypleurum alpinum, Crepis chrysantha* are encountered very rarely. Plants do not form dense tangled vegetation. They are encountered in separate groups or as single individuals. *Betula rotundifolia, Cotoneaster uniflorus* are rare among the shrubs, and *Juniperus sibirica* is individually encountered.
7. Population (*R. rosea* –*Trisetum altaicum–Deschampsia cespitosa*) occupy cold waterlogged coastal lake habitats, Ivanov ridge, near Maloye lake (50°18’36.9"N, 83°44’44.7"E), on the altitude of 2000–2100 m above sea level. Population lives at the northeastern shores of a permanent dammed lake in the close proximity of the water. PC is 15–25%. Tangles of *R. rosea* occupy a narrow coastal strip from the very edge of the water that is only 1.5–2 m wide. The vegetation cover is represented by separate plants or small groups of the communities, where *Сarex aterrima, Deschampsia cespitosa, Festuca borissii, Trisetum altaicum, Phleum alpinum, Swertia obtuse, Primula nivalis, Rhodiola algida, Sanguisorba alpina, Caltha palustris, Bistorta vivipara, Allium schoenoprasum, Gentiana algidа* are often encountered. *Salix lanata* and *S. rectijulis* are relatively rare. Among the herb layer, *R. rosea* is encountered relatively abundantly, the habitat conditions for the species can be considered close to optimal.
8. Population (*Salix lanata–Betula rotundifolia*–*R. rosea* ) occupies moderately humid bushy tundra habitats, in Ivanov ridge (50°19’36.4"N, 83°48’17.8"E). The communities with the participation of *R. rosea* are timed to the northwestern steep slopes on the altitude limit of 2100 – 2200 m above sea level. PC is 50-60 %. The herb layer is less abundant, *Carex aterrima, Trollius altaicus, Pedicularis oederi, Thalictrum alpinum, Macropodium nivale, Geranium albiflorum, Gagea serotina* are encountered. *Betula rotundifolia* tangles reach a height of 35-40 m, rarely 50 m. In this population, *R. rosea* grows in extreme habitat conditions, the population is aging, supported by inefficient vegetative reproduction. Gradually expanding, tangles of birches crowd them out from their habitats.
9. Population (*R. rosea* + *Achillea ledebourii– Veratrum lobelianum – Sanguisorba alpine*) occupies coastal and excessive wet meadows, constant verges of the streams, cedar-larch (*Pinus sibirica*, *Larix sibirica*) wood meadows on the altitude of 1700–1900 m (Fig. 3), Western Listvyaga, upper part of Repnaya river (49°21’06.0"N, 85°41’54.8"E). Excessive moisture and light shading throughout the growing season create unfavorable conditions for the development of *R. rosea*. The vegetation cover is relatively rich in species diversity, PC is 65–80%. Accompanying species are: *Alchemilla altaica, Primula nivalis, Carex curaica, С. aterrima* are often encountered in the community, while *Carex orbicularis, Cerastium davuricum, Bistorta vivipara, Trollius altaicus, Deschampsia cespitosa, Allium schoenoprasum, Myosotis scorpioides, Delphinium elatum, Caltha palustris* are rarely encountered. *R. rosea* is timed to areas in the form of narrow ribbons of 1.5 – 2 m width along the coasts. There are no shrubs. In rare cases, *Lonicera altaica* was noted along the coastline. *R. rosea* forms small clumps on areas bare from grass. In this habitat type generative individuals are dominating.
10. Population (*R. rosea* – *Dichodon cerastoides – Allium schoenoprasum*) occupy the shores of mountains streams, wells, drains which are of temporary natural due the process of snow melting patches of which the drying begins from the mid July, locations is Western Listvyaga ridge, in the vicinity of Schebnuiha mountain (49°21’54.0"N, 85°44’58.9"E), on the altitude limit of 2000–2200 m. *R. rosea* plants grow on the boulders covered by the thick moss cover. The vegetation cover in the phytocenosis is poorly expressed. PC is 30–40%. The following species are encountered in the community: *Primula nivalis, Carex orbicularis, C. aterrima, Bistorta vivipara, Pedicularis oederi, Deschampsia cespitosa, Micranthes punctata, Macropodium nivale, Sanguisorba alpina, Lagotis globosa, Gentiana algida*. Populations of *R. rosea* are of normal type, young, habitat conditions can be considered optimal.

**Fig. 4:**
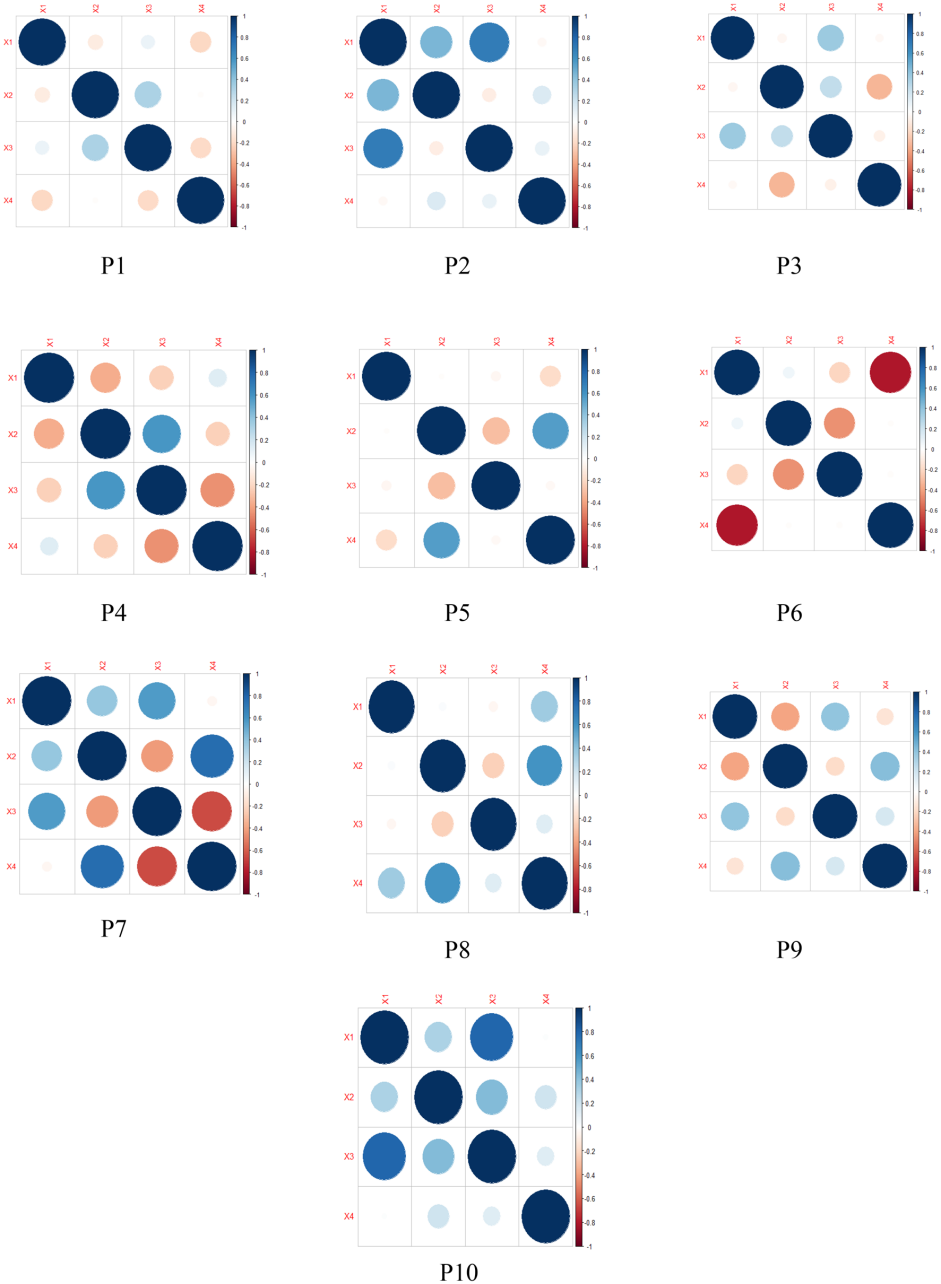
Correlation analysis of morphometric parameters and quantitative indicators of specimens within P1–P10 populations. Correlations with P < 0.05 are highlighted in color. The color indicates either positive (blue) or negative (red) correlation. (X1: Number of adult specimens per 1 m^2^; X2: Height of generative species at the time of bloomingg (cm); X3: The number of sprouts per a specimens (pcs); X4: Inflorescence diameter (cm))

Table 1 demonstrates the morphometric parameters of *R. rosea* in the surveyed populations of Kazakhstan Altai. According to the data obtained, the highest number of individuals per 1 m^2^ was observed in P10 (0.75), P7 (0.68) and P1 (0.56), a relatively low number per unit area was noted in P8 (0.18 ), P 6 (0.21) and in P3 (0.23). *R. rosea* populations with a high number of individuals per unit area, are composed of mostly undersized, multi-shoot shrubs having large flowers. In the population with a low abundance of *Rhodiola* specimens per unit area, tall individuals are observed, with loose, low-shoot bushes and relatively small flowers. *R. rosea* populations growing in habitats with a high number of accompanying species are multi-stemmed and have large flowers. Populations with a low number of individuals, are characterized by tall, crumbly, low-running bushes and relatively small inflorescences. This pattern is explained by the habitat conditions. As a rule in the forest belt and in tall grass communities the specimens of *R. rosea* are relatively tall (45–50 cm), have crumbly low-running bushes (6–10 pcs) and small inflorescences (3–4.5 cm). In open, poorly populated areas, and along the valleys of mountain streams, individuals are significantly undersized (20–25 cm), but have a multi-stem structure (30–50 pcs) and develop large flowers (5.2–6 cm) (Fig. 4).

**Table 1:** Quantitative and morphological indicators of *R. rosea* specimens from the study plots P1–P10

Quantative and morphological Values		Number of adult specimens per 1m2	Height of generative species at the time of blooming (cm)	The number of shoots per a specimen (pcs)	Inflorescence Diameter (cm)
P1	M	0.56	24.42	21.41	5.25
	SD	0.03	1.62	2.01	0.31
P2	M	0.32	48.33	15.22	4.20
	SD	0.01	1.28	1.61	0.26
P3	M	0.23	35.52	6.91	4.35
	SD	0.01	2.12	0.65	0.16
P4	M	0.28	26.66	20.32	3.63
	SD	0.01	2.91	1.71	0.18
P5	M	0.42	47.22	28.31	5.39
	SD	0.04	1.81	2.16	0.27
P6	M	0.21	38.30	12.27	4.23
	SD	0.01	1.21	1.63	0.15
P7	M	0.68	31.19	36.55	5.46
	SD	0.02	1.72	1.11	0.31
P8	M	0.18	49.29	8.12	3.81
	SD	0.01	2.33	0.69	0.25
P9	M	0.33	45.61	6.06	3.22
	SD	0.03	2.72	0.31	0.13
P10	M	0.75	32.30	37.33	4.81
	SD	0.03	1.44	2.82	0.33

### Phenology of *R. rosea* in East Kazakhstan

*R. rosea* begins to vegetate under snow cover starting from mid-May to mid-June in the studied area and when the snow melts, it begins to grow rapidly. Flowering period lasts from mid-June to late July. The fruits ripen from August to September. It should be noted that the seasonal rhythm of the species’ development depends on the height of the location of the population. The species begins to grow from mid-May in the upper limit of the green belt at an altitude of 1700–1900 m. *R. rosea* grows in the second half of June in the alpine belt at an altitude of 2200–2400 m. On average, the growing season lasts 4 months.

### Ontogenetic state *R. rosea*

The ontogenetic state of *R. rosea* was studied on Ivanov ridge, in the upper parts of Big Poperechka river (50°19’13.5"N, 83°45’11.0"E).

The species in the surveyed area reproduces predominantly in a vegetative way due to the division of rhizomes that are spreading by the melt water during the period of abundant snow melting, but in some locations seed renewal is noted. Seeds are small, elongated. The seed shape is curved-pin-shaped. The seeds surface is bare, longitudinally wrinkled. The seed scar is small, slightly protruding, basal, rounded. Seed color is from light brown to hazel. The length is 2.13 ± 0.16 mm, Cv = 15.7%; min–max – 1.65 – 2.78 mm, the width is 0.48–0.81 mm (0.59 ± 0.07 mm, Cv = 17.5%). The weight of 1000 seeds is 0.208–0.239 g. Once in the soil, ripened seeds undergo natural stratification over the next 7–8 months. Germination rate of *R. rosea* seeds in lab conditions at 18° C in three replications was 51%.

**Fig. 5:**
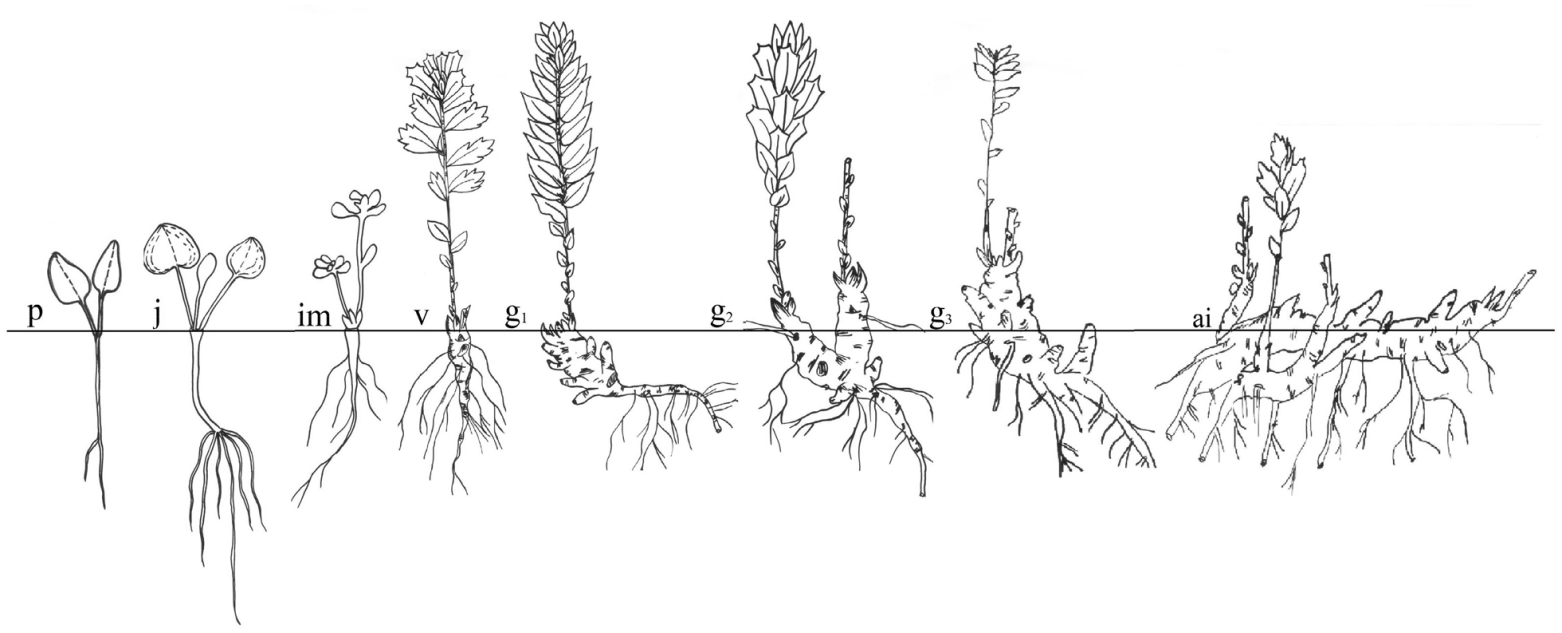
Ontogenesis of *R. rosea*: p: plantlets; j: juvenile; im: immature, v: virginile, g1: young generative, g2: mature generative; g3: old generative; a.i.: ageing individuals

Plantlets (Fig. 5(p)) appear at the end of May and beginning of June. Emergence of seeding is epigeous. The cotyledons are light green, bare, succulent 3,2±0.09 mm long, 1.8 ± 0.06 mm wide. The leaf blade is oval-ovoid, have short petiole up to 2.8 mm long, rounded at the apex, sharply tapered at the base turning into a short petiole. The hypocotyl is 3.2 ± 0.07 mm long, up to 1.1 ± 0.03 mm thick, pale green, the basal part is thickened, sharply passes into the embryonic (primary) root. The main root reaches 2.3 ± 0.08 cm by the time the cotyledons dry up. Cotyledons persist until mid or late July. In the second and the third months after germination of the seed, this age condition ends.

At the end of July and beginning of August the species pass into juvenile state (Fig. 5j). Plants in this phase are characterized by the formation of leaf rosette of 2.6 ± 0.04 leaves, the presence of a crown bud and 1–2 auxillary buds of an open type. The part of the growth sprout (rhizome) does not die off after the end of the vegetation season, but becomes perennial, from which the rhizome is subsequently formed. The seedlings end the juvenile phase at the age of 2–3 years old and less often.

Immature phase (Fig. 5(im)) is characterized by the growth of vegetative stems of normal type in the structure of medial sprout. The medial sprout of 7,2±0.12 cm height has 6,9 ± 0.21 pieces of natural leaves. The leaves are set by turn, the leaf blade is elongated-ellipsoid at the base and smoothly tapers into a short petiole. The crown and lateral buds of the growing sprout are of a closed type. The growth part of the rhizome is 2.4 ± 0.2 cm in length and 0.6 ± 0.03 cm in thickness. Rhizome branching is observed. The root system is well developed, in the horizontal projection is 2.8 ± 0.08 cm and in the vertical projection 12.1 ± 1.56 cm (deepened). In the primordial state, plants sustain on average 2 vegetation seasons. In the future, the plants move to the next age state.

Virginal phase (Fig. 5(v)) might be seen for 5–7 year and starts with the branching of the medial sprout of branching of the medial sprout of the rhizome with the development of a significant number of lateral sprouts of the first order with the development of stems on them. The plants in this age state are 14 ± 1.31 cm in height. The rhizome has 3,6 ± 0.07 pieces of stems of the first order. The medial sprout of the rhizome and some lateral sprouts are 6.2 ± 0.06 cm long at the base and 0.83 ± 1.2 cm across. The root system is well developed, 10.2 ± 1.8 cm in horizontal projection and 16 ± 2.2 cm in vertical projection. The primary root is about 1.8 ± 0.06 cm thick.

The plants start the young generative phase (Fig. 5(g1)) at the age of 8–11 years. Generative stems are formed in the sprout. Young plants usually generate 2.8 ± 0.06 pieces of generative stems with a depleted inflorescence of 1.8 ± 0.02 flowers and 8,2 ± 2.6 vegetative stems in their first two years. Plants at the age of 8–10. more often at 12 years begin to form generative stems on the sprouts of the rhizome of the first type. The rhizome of the horizontal projection is 3.2 ± 0.9 cm thick and 16.3 ± 0.8 cm long. Plants are dioecious. The flowers are unisexual, yellow, four-membered (less often five-membered), small (3.2 ± 0.06 mm long), collected in the final dense multi-flowered corymbose inflorescences. The number of flowers in an inflorescence are 6.3 ± 1.8 having a diameter of 4.8 ± 0.35 cm. The height of the plant is 30 ± 1.8 cm. In this age state, stems begin to form on the sprouts of second-order rhizomes. This age-related condition ends at 18–22 years.

The mature generative individuals (Fig. 5(g2)) include plants of 22–30 years, characterized by a powerful development of 43 ± 2.1 cm in height. In this state there is an intensive development of the stems on the medial sprout of the rhizome and sprouts of the first and second types. Such individuals have 35 ± 3.6 pcs of generative and 42 ± 2.8 pcs of vegetative stems. The inflorescence has 10.6 ± 1.7 flowers being 5.2 ± 0.35 cm in diameter. In this phase abundant flowering and fruiting and clone formation was observed. Particulation and clone formation are observed.

The plants start old generative phase (Fig. 5(g3)) at the age of 30–40 years. In this state, there is a noticeable predominance of the vegetative stems up to 68 ± 3.9 pcs and the formation of a significant number of weakened generative stems 52 ± 2.8 pcs. The inflorescence is composed usually of 6.2 ± 0.12 flowers, having 3.8 ± 0.35 cm in diameter. We observed here the beginning of the rhizome sprout decline and the formation of extensive foci of the main root necrosis and medial sprout of the rhizome. Also, mass dying of rhizomes’ sprout of the first order is typical in this age phase.

Ageing individuals (Fig. 5(a.i.)) are very rare. It is pretty hard to define the age of this species. According to our data, specimens start this phase at the age of 50-55 years. In this state, extensive foci of necrosis appear almost along the entire length of the main rhizome and its disintegration into separate girders can be seen. There are frequent cases of dying of the adventitious roots and of the first-order rhizome sprouts. The bushes easily break up into 3-6 clones, form new plants and spread over the population area.

### Floristic composition of the *R. rosea* communities

As a result of data processing of our field studies and herbarium collections, in the floristic composition of *R. rosea* communities we registered 140 species belonging to 39 families and 104 genera, which is 14% of the Altai highland flora (AHF), where 996 species of vascular plants from 325 genera and 80 families are registered (Table 2). Herbarium collections are kept in the herbarium of the NUR and Alt.

**Table 2:** Floristic composition of the studied *R. rosea* study plots

№	The species name	Occurrence of species	Life forms (Raunkiaer 1934)	Life forms (Serebryako, 1962)	Ecological groups	Area
*Amaryllidaceae*						
1.	*Allium schoenoprasum* L.	III	C	Bbp	HP	euras.
2.	*Allium flavidum* Ledeb.	I	C	Bbp	MP	as.
*Apiaceae*						
3.	*Neogaya simplex* (L.) Meisn.	II	H	Tp	P	euras.
4.	*Angelica archangelica* L.	III	H	Tp	М	as.
5.	*Angelica decurrens* (Ledeb.) B.Fedtsch.	II	H	Tp	М	as.
6.	*Bupleurum multinerve* DC.	II	H	Tp	MP	as.
7.	*Sajanella monstrosa* (Stephan ex Schult.) Soják	III	H	Tp	P	as.
8.	*Schulzia crinita* (Pall.) Spreng.	IV	H	Tp	P	as.
*Asteraceae*						
9.	*Aster alpinus* L.	II	H	Tp	X	holarc.
10.	*Achillea millefolium* L.	II	H	Lrp	М	holarc.
11.	*Saussurea alpina* (L.) DC.	II	H	Lrp	P	holarc.
12.	*Senecio nemorensis* L.	I	H	Brp	М	euras.
13.	*Solidago virgaurea* L.	III	H	Brp	М	euras.
14.	*Hieracium virosum* Pall.	II	H	Tp	MX	euras.
15.	*Crepis chrysantha* (Ledeb.) Turcz.	I	H	Srp	P	euras.
16.	*Saussurea latifolia* Ledeb.	II	H	Lrp	М	as.
17.	*Achillea ledebourii* Heimerl	IV	H	Brp	М	as.
18.	*Frolovia frolowii* (Ledeb.) Raab-Straube	II	H	Tp	MP	as.
19.	*Leuzea carthamoides* (Willd.) DC.	II	H	Tp	MP	as.
20.	*Doronicum altaicum* Pall.	IV	H	Tp	P	as.
*Berberidaceae*						
21.	*Berberis sibirica* Pall.	I	N	S	MPt	cent.as
*Betulaceae*						
22.	*Betula glandulosa* Michx. (=*B. rotundifolia* Spach)	III	N	S	P	euras.
*Boraginaceae*						
23.	*Myosotis scorpioides* L.	II	H	Brp	H	holarc.
24.	*Myosotis sylvatica* Ehrh. ex Hoffm.	I	H	Brp	М	euras.
25.	*Eritrichium villosum* (Ledeb.) Bunge	I	H	Tp	P	euras.
*Brassicaceae*						
26.	*Cardamine macrophylla* Willd.	II	C	Lrp	HP	as.
27.	*Macropodium nivale* (Pall.) W.T.Aiton	IV	H	Tp	P	as.
*Campanulaceae*						
28.	*Campanula cervicaria* L.	I	H	Tp	М	euras.
*Caprifoliaceae*						
29.	*Patrinia sibirica* (L.) Juss.	II	H	Tp	XPt	as.
30.	*Valeriana dubia* Bunge	I	C	Brp	MX	as.
31.	*Lonicera caerulea subsp. altaica* (Pall.) Gladkova	II	M	S	MP	as.
*Caryophyllaceae*						
32.	*Dichodon cerastoides* (L.) Rchb.	I	Ch	Srp	P	holarc.
33.	*Cherleria biflora* (L.) A.J.Moore & Dillenb.	I	Ch	Tp	P	holarc.
34.	*Sabulina verna* (L.) Rchb.	II	H	Tp	P	holarc.
35.	*Sagina saginoides* (L.) H.Karst.	II	Ch	Tp	P	holarc.
36.	*Dianthus superbus* L.	II	H	Lrp	М	euras.
37.	*Cerastium davuricum* Fisch. ex Spreng.	III	H	Tp	HP	as.
38.	*Silene bungei* Bocquet	I	H	Tp	P	as.
*Crassulaceae*						
39.	*Hylotelephium telephium* (L.) H.Ohba	IV	H	Brp	М	euras.
40.	*Phedimus hybridus* (L.) Hart	III	Ch	Srp	XPt	as.
41.	*Hylotelephium ewersii* (Ledeb.) H.Ohba	II	Ch	Brp	MPt	as.
42.	*Rhodiola algida* (Ledeb.) Fisch. & C.A.Mey.	IV	Ch	Tp	P	Alt.
*Cupressaceae*						
43.	*Juniperus communis* var. *saxatilis* Pall. (=*J. sibirica* Burgsd.)	II	N	S	MP	euras.
*Cyperaceae*						
44.	*Eriophorum angustifolium* Honck.	II	H	Lrp	HP	holarc.
45.	*Carex capillaris* L.	V	H	HS	H	euras.
46.	*Carex aterrima* Hoppe	IV	H	Brp	HP	euras.
47.	*Carex pediformis* var. *macroura* (Meinsh.) Kük.	II	H	Srp	MX	euras.
48.	*Carex curaica* Kunth	I	H	Brp	H	as.
49.	*Carex stenocarpa* Turcz. ex V.I.Krecz.	IV	H	Srp	P	as.
50.	*Carex altaica* (Gorodkov) V.I.Krecz.	I	C	Lrp	HP	Alt.
*Ericaceae*						
51.	*Vaccinium myrtillus* L.	II	Ch	Ds	MP	holarc.
*Euphorbiaceae*						
52.	*Euphorbia pilosa* L.	IV	H	Tp	XPt	tur.
*Fabaceae*						
53.	*Hedysarum neglectum* Ledeb. (=*Hedysarum austrosibiricum* B.Fedtsch.)	III	H	Tp	М	euras.
54.	*Oxytropis purpurea* (Bald.) Markgr.	II	H	Tp	М	euras.
55.	*Trifolium lupinaster* L.	IV	H	Srp	MX	euras.
56.	*Hedysarum theinum* Krasnob.	III	H	Tp	MP	as.
57.	*Thermopsis alpina* (Pall.) Ledeb.	II	H	Lrp	P	as.
58.	*Oxytropis alpina* Bunge	III	H	Tp	P	Alt.
*Gentianaceae*						
59.	*Swertia obtusa* Ledeb.	III	H	Lrp	М	as.
60.	*Gentiana algida* Pall.	II	H	Brp	P	as.
61.	*Gentiana grandiflora* Laxm.	I	Ch	Tp	P	as.
*Geraniaceae*						
62.	*Geranium albiflorum* Ledeb.	II	H	Brp	М	as.
*Juncaceae*						
63.	*Luzula spicata* (L.) DC.	I	H	Brp	P	holarc.
*Lamiaceae*						
64.	*Dracocephalum ruyschiana* L.	II	C	Srp	М	euras.
65.	*Dracocephalum peregrinum* L.	I	H	Tp	XPt	as.
66.	*Dracocephalum grandiflorum* L.	III	H	Srp	MP	as.
*Liliaceae*						
67.	*Gagea serotina* (L.) Ker Gawl. (=*Lloydia serotina* (L.) Rchb.)	II	C	Bp	P	holarc.
*Lycopodiaceae*						
68.	*Diphasiastrum alpinum* (L.) Holub	II	Ch	Cm	P	holarc.
*Melanthiaceae*						
69.	*Veratrum lobelianum* Bernh.	III	H	Brp	М	as.
*Montiaceae*						
70.	*Claytonia joanneana* Schult.	I	H	Tp	P	as.
*Onagraceae*						
71.	*Epilobium palustre* L.	II	H	Srp	HP	holarc.
72.	*Epilobium angustifolium* L.	I	H	Tp	М	holarc.
*Orobanchaceae*						
73.	*Pedicularis oederi* Vahl	III	H	Tp	P	holarc.
74.	*Pedicularis violascens* Schrenk	I	H	Tp	XPt	as.
75.	*Pedicularis amoena* Adams ex Steven	II	H	Tp	P	as.
76.	*Pedicularis achilleifolia* Stephan ex Willd.	I	H	Tp	XPt	as.
*Papaveraceae*						
77.	*Papaver nudicaule* L.	III	H	Tp	MP	as.
78.	*Papaver croceum* Ledeb.	I	H	Tp	P	as.
*Pinaceae*						
79.	*Abies sibirica* Ledeb.	I	M	T	М	euras.
80.	*Larix sibirica* Ledeb.	I	M	T	М	euras.
81.	*Picea obovata* Ledeb.	I	M	T	М	euras.
82.	*Pinus sibirica* Du Tour	I	M	T	М	euras.
*Plantaginaceae*						
83.	*Veronica densiflora* Ledeb.	I	H	Lrp	P	as.
*Poaceae*						
84.	*Deschampsia cespitosa* (L.) P.Beauv.	IV	H	Brp	H	cosm.
85.	*Anthoxanthum monticola* (Bigelow) Veldkamp	I	H	Srp	М	cosm.
86.	*Festuca rubra* L.	III	H	Brp	HP	holarc.
87.	*Poa alpigena* Lindm.	II	C	Lrp	М	holarc.
88.	*Festuca borissii* Reverd.	IV	H	Brp	MP	holarc.
89.	*Calamagrostis purpurea* (Trin.) Trin.	II	H	Lrp	H	euras.
90.	*Elymus repens* (L.) Gould	II	C	Lrp	М	euras.
91.	*Alopecurus pratensis* L.	II	C	Srp	М	euras.
92.	*Dactylis glomerata* L.	III	H	Brp	М	euras.
93.	*Helictochloa hookeri* (Scribn.) Romero Zarco	II	H	Brp	MX	euras.
94.	*Phleum alpinum* L.	II	H	Brp	P	euras.
95.	*Poa sibirica* Roshev.	I	H	Brp	MP	as.
96.	*Paracolpodium altaicum* (Trin.) Tzvelev	I	H	Lrp	P	as.
97.	*Trisetum altaicum* Roshev.	II	H	Brp	P	as.
98.	*Festuca kryloviana* Reverd.	IV	H	Brp	P	as.
99.	*Poa attenuata* Trin.	III	H	Brp	MX	Alt.
100.	*Koeleria altaica* (Domin) Krylov	I	H	Brp	P	Alt.
*Polygonaceae*						
101.	*Bistorta vivipara* (L.) Delarbre	III	H	Brp	HP	holarc.
102.	*Oxyria digyna* (L.) Hill	II	H	Lrp	MPt	holarc.
103.	*Koenigia alpina* (All.) T.M.Schust. & Reveal	II	H	Tp	М	euras.
104.	*Rumex acetosa* L.	III	H	Tp	М	euras.
105.	*Rumex scutatus* L.	II	H	Tp	М	euras.
106.	*Bistorta elliptica* (Willd. ex Spreng.) V.V.Petrovsky, D.F.Murray & Elven	III	H	Brp	P	euras.
*Primulaceae*						
107.	*Primula nivalis* Pall.	I	H	Brp	HP	as.
*Ranunculaceae*						
108.	*Ranunculus lapponicus* L.	II	H	Lrp	H	holarc.
109.	*Caltha palustris* L.	III	H	Brp	H	holarc.
110.	*Thalictrum alpinum* L.	I	H	Srp	P	holarc.
111.	*Delphinium elatum* L.	IV	H	Srp	М	euras.
112.	*Thalictrum flavum* L.	II	H	Brp	М	euras.
113.	*Aconitum septentrionale* Koelle	III	H	Tp	М	euras.
114.	*Trollius altaicus* C.A.Mey.	II	H	Srp	М	as.
115.	*Anemonastrum narcissiflorum* (L.) Holub	II	H	Srp	MX	as.
116.	*Aquilegia flabellata* Siebold & Zucc.	IV	H	Srp	MX	as.
117.	*Callianthemum alatavicum* Freyn	II	H	Srp	P	as.
118.	*Trollius lilacinus* Bunge	I	H	Brp	P	as.
119.	*Aconitum apetalum* (Huth) B.Fedtsch.	III	H	Brp	MP	Alt.
120.	*Aconitum glandulosum* Rapaics	II	H	Bbp	MP	Alt.
121.	*Ranunculus altaicus* Laxm.	IV	H	Brp	P	Alt.
*Rosaceae*						
122.	*Dasiphora fruticosa* (L.) Rydb.	II	N	S	М	holarc.
123.	*Spiraea media* Schmidt	II	N	S	М	euras.
124.	*Alchemilla altaica* Juz.	IV	H	Srp	М	euras.
125.	*Cotoneaster uniflorus* Bunge	II	N	S	P	euras.
126.	*Sibbaldia procumbens* L.	III	Ch	Lrp	P	as.
127.	*Dasiphora glabrata* (Willd. ex Schltdl.) Soják	II	N	S	P	as.
128.	*Dryas oxyodonta* Juz.	III	Ch	Ds	P	as.
129.	*Sanguisorba alpina* Bunge	III	H	Tp	P	as.
130.	*Sibiraea laevigata* (L.) Maxim.	II	N	S	MX	Alt.
*Rubiaceae*						
131.	*Galium boreale* L.	II	H	Lrp	М	holarc.
*Salicaceae*						
132.	*Salix lanata* L.	II	N	S	P	holarc.
133.	*Salix turczaninowii* (Laksch.)	II	N	Ds	P	euras.
134.	*Salix rectijulis* Ledeb. ex Trautv.	II	N	S	P	as.
*Saxifragaceae*						
135.	*Saxifraga sibirica* L.	I	H	Brp	HP	as.
136.	*Micranthes punctata* (L.) Losinsk.	II	H	Tp	HP	as.
137.	*Bergenia crassifolia* (L.) Fritsch	II	H	Lrp	MPt	as.
*Urticaceae*						
138.	*Urtica dioica* L.	I	H	Lrp	М	euras.
*Violaceae*						
139.	*Viola biflora* L.	II	H	Srp	MP	holarc.
140.	*Viola altaica* Ker Gawl.	III	H	Tp	P	as.
Occurrence of species: I – 0–20%, II – 21–40%, III – 41–60%, IV – 61–80%, V – 81–100% Life forms according to Raunkiaer (1934): M - Mesophanerophytes, N - Nanophanerophytes, Ch - Chamaephyts, H - Hemicryptophytes, C - Cryptophytes Life forms according to Serebryakov, (1962): T – tree; S – shrub; Hs – half-shrub; Ds – dwarfshrub; Lrp – long rhizomatous plant; Srp – short rhizomatous plants; Bbp – bulbotuberiferous plants; Bp – bulbous plants; Tp –taproot plants; Brp – brushy root plants; Tsp – tussock plants; Cm – club-moss Ecological groups of plants in relation to the temperature, moisture and ston nature of the substrate: H - hygrophytes, HP - hygropsychrophytes, GM - hygromesophytes, M - mesophytes, MX - mesoxerophytes, MP - mesopsychrophytes, X - xerophytes, XPt - xeropetrophyte, P - psychrophytes, MPt - mesopetrophytes The groups of floral elements: cosm. - cosmopolitan; holarc. - Holarctic; euras. - Eurasian; as. - Asian; tur. - Turanian; Mtr - Mediterranean; Alt - Altai (endemics of the Altai-Sayan botanical-geographical province)						

**Fig. 6:**
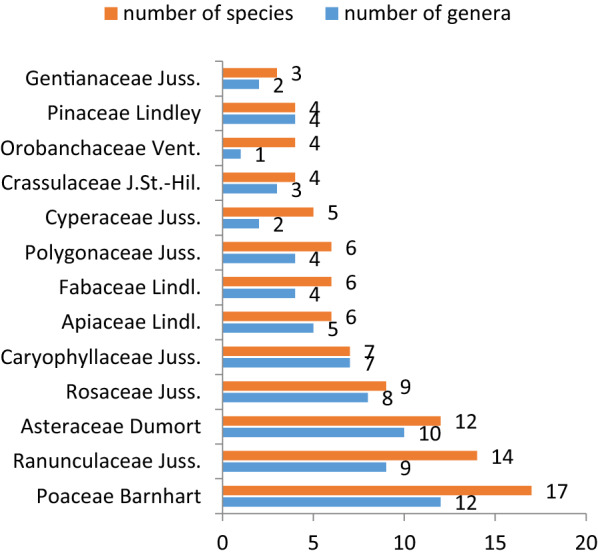
Leading families with the number of genera and species within the composition of the *R. rosea* habitats

The systematic analysis of the floristic composition of *R. rosea* communities showed that leading families in terms of the species frequencies are *Poaceae* Barnhart (12%), *Ranunculaceae* Juss. (10 %), *Asteraceae* Bercht. & J.Presl (9 %), *Rosaceae* Juss. (7%), *Caryophyllaceae* Juss. (5%), *Apiaceae* Lindl. (4%), *Fabaceae* Lindl. (4%) and *Polygonaceae* Juss. (4%) (Fig. 6). They account for 77 (55%) species of the flora composition.

**Fig. 7:**
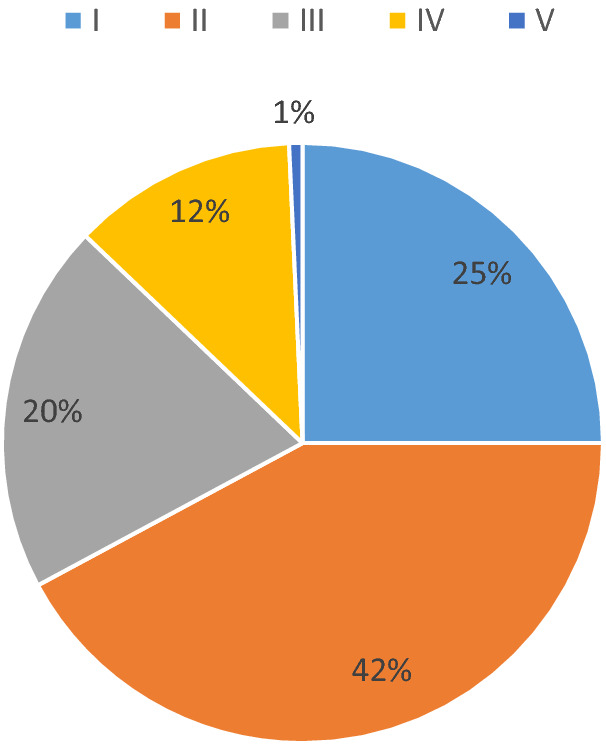
Species composition in the flora of *R. rosea* habitats

The most common 17 species in the floristic composition of the habitats are (occurrence IV–V): *Schulzia crinita*, *Achillea ledebourii*, *Doronicum altaicum*, *Macropodium nivale*, *Hylotelephium telephium*, *Rhodiola algida*, *Carex capillaris*, *C. aterrima*, *C. stenocarpa*, *Euphorbia pilosa*, *Trifolium lupinaster*, *Deschampsia cespitosa*, *Festuca borissii*, *F. kryloviana*, *Delphinium elatum*, *Aquilegia flabellata*, *Ranunculus altaicus*, *Alchemilla altaica*, 35 species are rather rare (occurrence I). The number of species in the composition of the flora range from 21 to 40% (II) - 59 species and from 41 to 60% (III) - 28 species (Fig. 7).

**Fig. 8:**
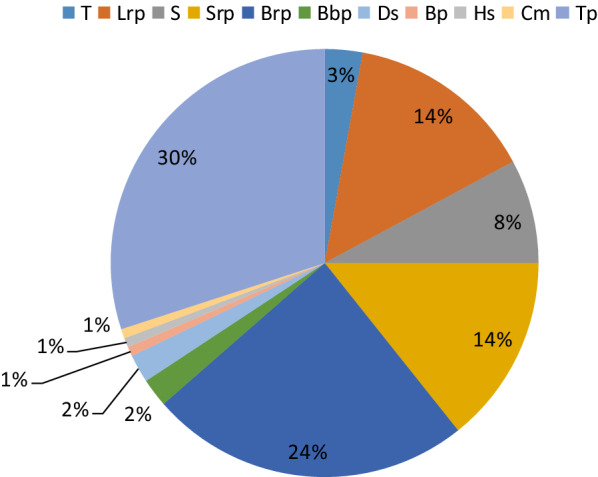
Life forms composition the floristic composition of the habitats according to Serebryakov (1962): T, tree; S, shrub; Hs, half-shrub; Ds, dwarfshrub; Lrp, long rhizomatous plant; Srp, short rhizomatous plants; Bbp, bulbotuberiferous plants; Tp, taproot plants; Brp, brushy root plants; Cm, club-moss

In the floral composition of *R. rosea* communities, all species are perennials. The analysis of life forms showed that tap root plants (30%), brushy root plants (34%), short rhizomatous plants (14%), long rhizomatous plants (14), shrubs (7%) prevail in communities with the participation of *R. rosea* (Fig. 8).

**Fig. 9:**
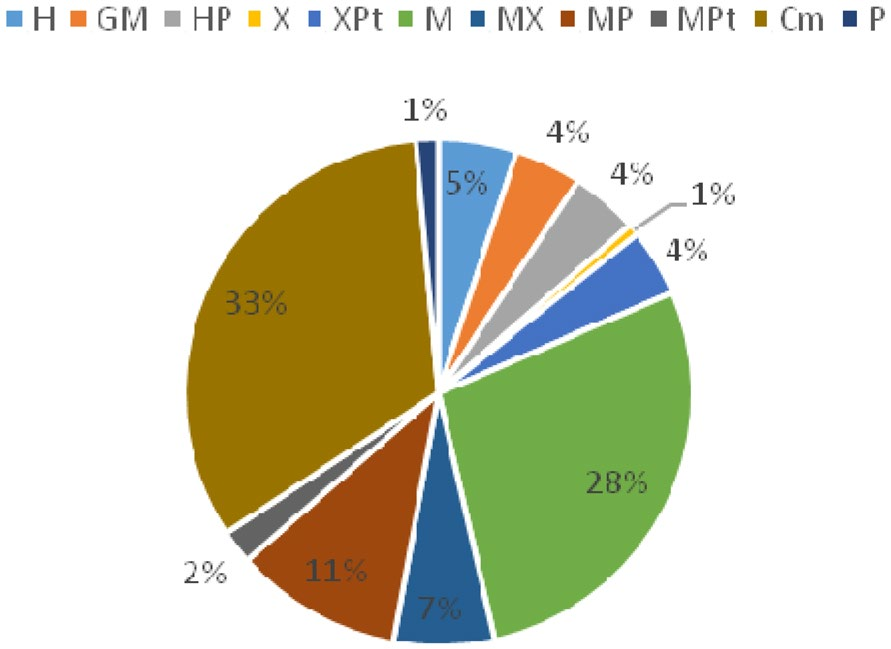
Composition of the ecological groups in the flora of the *R. rosea* habitats: H, hygrophytes; GM, hygromesophytes; HP, hygropsychrophytes; X, xerophytes; XPt, xeropetrophyte; MX, mesoxerophytes; MP, mesopsychrophytes; X, xerophytes; XPt, xeropetrophyte; P, psychrophytes; MPt,  mesopetrophytes

Data on the distribution of species by ecological groups, according to the classification of Kuminova A.V. (1960), showed that psychrophytes (32%), mesophytes (28%), mesopsychrophytes (11%) and mesoxerophytes (7%) are dominating in the floral composition of *R. rosea* communities (Fig. 9).

**Fig. 10:**
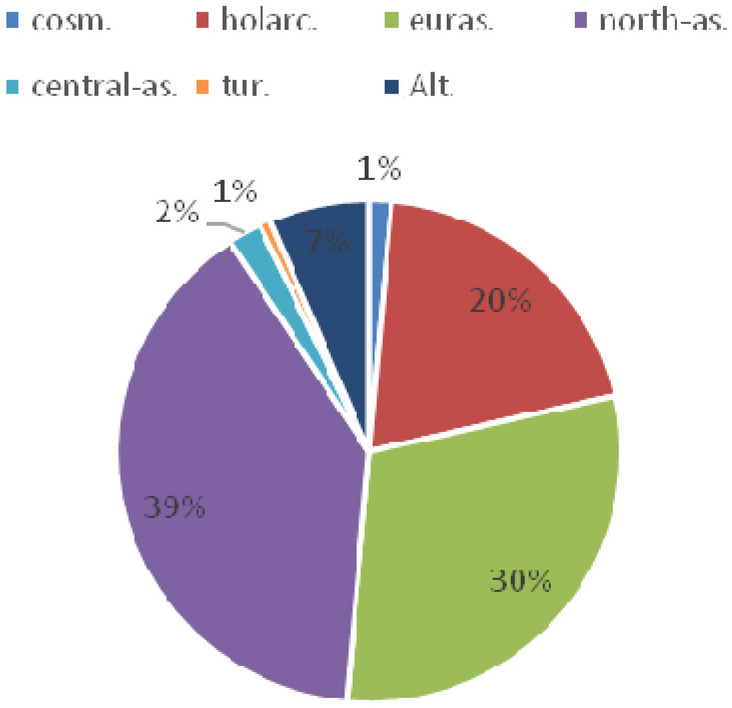
Chorological analysis of *R. rosea* habitats: cosm., cosmopolitan; holarc., Holarctic; euras., Eurasian; north-as., north Asian; tur., Turanian; Alt, Altai (endemics of the Altai-Sayan botanical-geographical province)

The chorological analysis shows that the Asian group (39%), the Eurasian group (30%), and the Holarctic species (20%) are most abundant (Fig. 10).

**Fig. 11:**
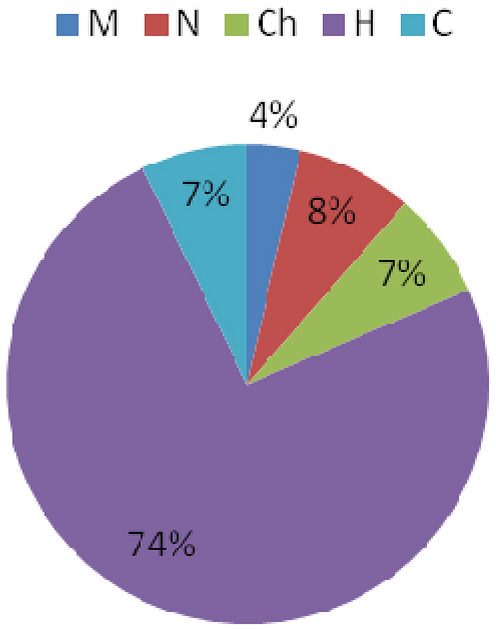
Composition of the life forms in the flora *R. rosea* habitats (Raunkiaer 1934): M, Mesophanerophytes; N, Nanophanerophytes; Ch, Chamaephyts; H, Hemicryptophytes; C, Cryptophytes

Analysis of the life forms by Raunkiaer (1934) showed that in communities with *R. rosea*, the vast majority of species are Hemicryptophytes (74%), a small amount of species are Mesophanerophytes (7%), Nanophanerophytes (8%), Chamaephyts (7%). Cryptophytes account for 4% (Fig. 11).

## Discussion

In the studies of Vedernikova and Nikandrova (2000) interesting differences was found between two groups of populations *R. rosea*, when studying interpopulation variation which are populations timed to the mountain tundra belt on the one hand, and populations timed to near-snowy lawns and river meadows on the other hand. In the second group of populations, a tendency towards a clear predominance of features which characterize the general habit of the plant (height of sprouts, their number, leaf size, etc.) is noted. Therefore, these environmental conditions can be considered optimal for *R. rosea*. Our studies also confirmed this theory. In open, sparsely populated areas and along the valleys of mountain streams, individuals are significantly stunted (20–25 cm), but have multi-stemmed bushes (30–50 pcs) and large inflorescences (5.2–6 cm in diameter). The height of *R. rosea* individuals in the examined populations ranges from 24.42 to 49.29 cm, the diameter of the inflorescence varies between 3.21 and 5.25 cm. These parameters are within the normal range for the species (Eggli 2003).

In the studied region, *R. rosea* is found in different ecological conditions and altitudinal ranges of 1700–2400 m (from the upper limit of the forest belt to the tundra belt of the mountains). The wide range of habitats of the species is also confirmed by other researchers (Allen et al. 2014; Cuerrier and Ampong-Nyarko 2014; Fu et al. 2001).

Based on our study we can confirmed that *R. rosea* is present in the Southwestern and Southern Altai of the studied region, as well as on the Tarbagatai ridge. When reviewing the herbarium collections of AA, one herbarium collection was found from the territory of the Kalbinsky ridge (Koktau mountains under the ridge of rocks 1440 m, singly, Snegirev V., 31. V. 1994 (AA)), but our studies did not confirm this location of the species. Perhaps an error could have been made in labeling, or this collection may be evidence of the former distribution of the species preserved in the relict complex “Sinegorskaya fir Grove” (Myrzagalieva 2006).

*R. rosea* plants are known to be long-lived perennials, but maximum ages in wild populations have not been studied (Brinckmanna et al. 2021). According to Nekratova and Nekratov (2005), the maximum age of *R. rosea* is 80 years. Our researches allowed us to establish the maximum age of *R. rosea* in the studied region at 55 years, after which the bushes easily disintegrate into 3–6 clones, forming new plants and spreading over the population area.

According to Revushkin (1988), 996 species of vascular plants from 325 genera and 80 families were registered in the AHF, the species diversity of the floral composition of *R. rosea* communities was 140 species, which is 14% of the AHF flora. The composition based on the 10 most frequent families in terms of their quantities is almost identical to the composition of the leading families in AHF. However, their arrangement in descending order is somewhat different, which is due to the allocation of *R. rosea* to the redivided ecological groups. Communities with *R. rosea* are dominated by single and two species families, which characterizes the AHF flora (Sofronov et al. 2016), the Western Sayan (Kamelin 2016) and the arctic floras which develop in extreme habitat conditions and indicates the complicacy of the floral genesis process (Sofronov et al. 2016). The analysis of the genera spectrum accords well with the composition of the AHF leading genera (Revushkin 1988), where *Carex*, *Aconitum*, *Dracocephalum*, *Festuca*, *Pedicularis*, *Poa*, *Salix* genera prevail.

According to Revushkin (1988) it is enough to consider in the ecological analysis of highlands the humidity and the type of the substrate. No need of taking into account the temperature, the salinity or the soil fertility. However, we consider that the classification of ecological groups presented by Kuminova (1960) is well applicable for the highland flora analysis and the temperature regime is the crucial feature of the highlands. Despite that, we agree that it is inappropriately to allocate groups due to their salinity and soil fertility. The data showed that the composition of the flora is dominated by psychrophytes, mesophytes, mesopsychrophytes and mesoxerophytes. These groups account for 109 (78%) species of the total floristic composition of the *R. rosea* communities. In general, this distribution of species by ecological groups is considered characteristic for the flora of the highlands.

The percentage of the chorological groups differs somewhat from the AHF, however, the leading groups nevertheless converge. It should be noted that a significant excess of the Eurasian group was detected, (30%) compared to the AHF (14.4%), and the participation of Asian species is in the opposite way lower (39%) than the AHF (62%). The distribution of species by life forms is in good agreement with the AHF flora.

## Conclusions

In general, the state of *R. rosea* populations in the studied region should be considered satisfactory. In the surveyed populations anthropogenic influence was not observed, apparently because of difficult accessibility of the habitats. The main limiting factor is the low competitiveness of the species within the communities. When the habitats are overgrown with herbaceous-grass vegetation, populations of *R. rosea* are forced to gradually migrate to less overgrown areas, or fall out of the phytocenosis. All surveyed populations are maintained mainly by vegetative reproduction. Seed renewal is low as a result of seedlings dying in the early stages of development due to extreme habitat conditions.

## Data Availability

The data used and analyzed in this study can be provided from the respective author for scientifc, non-proft purpose.

## Abbreviations

PC: Projective cover
CIS: Commonwealth of Independent States
WHO: World Health Organization
POWO: Plants of the World Online
AHF: Altai highland flora
